# Unlocking ESCC Progression: CCL15-CCR1 Axis Activates AKT/ERK1/2/c-Jun/CDK2 Pathway

**DOI:** 10.7150/jca.113925

**Published:** 2025-07-04

**Authors:** Shengliang He, Yunjiu Gou, Qizhou Bo, Dacheng Jin, Songchen Han, Hui Cai

**Affiliations:** 1 The First Clinical Medical College of Lanzhou University, Lanzhou University, 730000, China.; 2 NHC Key Laboratory of Diagnosis and Therapy of Gastrointestinal Tumor, Gansu Provincial Hospital, 730000, China.; 3 Key Laboratory of Molecular Diagnostics and Precision Medicine for Surgical Oncology in Gansu Province, Gansu Provincial Hospital, 730000, China.; 4 Gansu Provincial Hospital, Lanzhou 730000, Gansu, China.

**Keywords:** CCR1, ESCC, CCL15, CDK2, cell proliferation and migration

## Abstract

Globally recognized as a major oncological concern, esophageal squamous cell carcinoma (ESCC) exhibits a high incidence rate among malignancies originating from the digestive system's epithelial lining. Tumor cells can secrete chemokine (C-C motif) ligand 15 (CCL15) to recruit C-C motif chemokine receptor 1 (CCR1)-positive macrophages, thereby facilitating immune evasion and promoting tumor progression. However, it remains unclear whether CCL15 secreted by tumor cells can also act directly on CCR1 expressed on the same tumor cells to exert tumor-regulatory effects. Here, we discovered that both CCL15 and CCR1 are significantly overexpressed in tumor tissues of patients diagnosed with ESCC. Moreover, baseline expression levels of CCR1 and CCL15 in ESCC cell lines EC109, TE-1, and KYSE150 were markedly higher than those in the normal esophageal epithelial line HET-1A. *In vitro* experiments demonstrated that recombinant human CCL15 (rhCCL15) significantly enhanced proliferation, migration, and invasion of EC109 and TE-1. While knockdown of CCL15 or CCR1 using lentiviral approaches markedly inhibited the proliferation and migration. Notably, CCR1 knockdown reversed the tumor-promoting effects of rhCCL15 on ESCC cells. Subsequent immunofluorescence co-localization and co-immunoprecipitation confirmed a direct interaction between CCR1 and CCL15. Mechanistically, PCR array analysis identified cyclin dependent kinase 2 (CDK2) as a downstream effecter of CCL15-CCR1 axis. Furthermore, through transcription factor prediction, protein-protein interaction (PPI) database analysis, and ChIP-qPCR assays, we demonstrated that CDK2 transcription is activated *via* CCL15-CCR1-mediated phosphorylation of c-Jun through the AKT/ERK1/2 pathway. In addition, we conducted a drug screening targeting CCR1 and identified Jervine as a potential CCR1 degrader. In summary, this study uncovers the relationship between CCL15-CCR1 axis and ESCC progression and provides insights into potential therapeutic strategies targeting this pathway.

## 1. Introduction

As a significant clinical entity in oncology, esophageal carcinoma imposes a substantial global disease burden, characterized by high epidemiological prevalence and poor survival outcomes 1. In China, approximately 80% of esophageal cancer cases are classified as ESCC 2. ESCC ranks seventh among cancer-related causes of death, with an estimated 600,000 deaths reported in 2020 alone. Despite remarkable advancements in diagnostic modalities and therapeutic interventions over the past decade, the incidence of ESCC remains high, and the 5-year survival rate continues to fall below 20%, posing ongoing clinical challenges 3. Thus, investigating the potential molecular mechanisms of ESCC and identifying effective therapeutic strategies remain pressing priorities.

CCL15 is a 113-amino acid chemokine that is upregulated in various tumor types 4, 5. Targeted deletion of CCL15 has been shown to significantly attenuate tumor progression 4, 6, 7. Previous studies have demonstrated that tumor-derived CCL15 promotes the progression of hepatocellular carcinoma by modulating immune cell recruitment and activation, thereby maintaining an immunosuppressive microenvironment 4. In colorectal cancer, CCL15 facilitates lung metastasis by recruiting neutrophils, and promotes liver metastasis by attracting myeloid cells 8. Moreover, CCL15 is also important in regulating the progression of melanoma and pancreatic cancer. Mechanistically, CCL15 interacts with multiple chemokine receptors, especially CCR1, to recruit macrophages, neutrophils, and lymphocytes, thereby regulating tumor growth 6, 9-11. As a G protein-coupled receptor superfamily constituent, CCR1 mediates oncogenic processes through dual regulation of cellular proliferation and apoptotic pathways, primarily via PI3K/AKT and ERK1/2 signaling cascades 12-15. These studies underscore the critical function of CCL15-CCR1 axis in executing crosstalk between tumor and immune cells to promote tumorigenesis. However, whether CCL15 can directly activate CCR1 expressed by tumor cells themselves to drive tumor progression remains unclear. Additionally, the specific contribution of the CCL15-CCR1 axis in ESCC has not been fully elucidated.

In this study, we identified that CCL15 and CCR1 are highly expressed in patient-derived ESCC tissues, with elevated baseline expression observed in ESCC cell compared to esophageal epithelial cells. Based on these findings, we hypothesized that the tumor-intrinsic CCL15-CCR1 axis contributes to ESCC progression. Through rhCCL15 treatment and knockdown of CCL15 and CCR1, we confirmed both *in vitro* and *in vivo* that the CCL15-CCR1 drives ESCC progression. Further analysis revealed that CCL15-CCR1 activates CDK2 transcription through AKT/ERK1/2-dependent phosphorylation of c-Jun at Ser63. Additionally, we conducted a small-molecule screen for CCR1 to seek potential drugs that could be used to mitigate the progression of ESCC. Collectively, this study provides mechanistic insights into ESCC pathogenesis and identify novel therapeutic targets for precision oncology approaches.

## 2. Materials and Methods

### 2.1 Cell culture

Human ESCC cell lines TE-1, EC109 and KYSE150 were purchased from Wuhan PriCella Biotechnology. TE-1 and EC-109 were cultured in RPMI-1640 medium supplemented with 10% fetal bovine serum (FBS) and 1% penicillin-streptomycin. All cell culture reagents were obtained from Gibco (USA). Cells were stayed at 37°C in a humidified incubator with 5% CO_2_. Cultures were grown in T25 flasks and passaged when they reached approximately 85% confluency.

### 2.2 Cell proliferation assay

For proliferation assays, 5 × 10³ cells per well were seeded and treated with recombinant human CCL15 (rhCCL15) for 48 hours. Following treatment, 10 μL of Cell Counting Kit-8 (CCK-8) reagent (Beyotime, China) was added to each well. After 2 hours of incubation, absorbance was measured at 450 nm to evaluate cell viability and proliferation.

### 2.3 Lentiviral packaging and gene silencing

shRNA sequences targeting genes of interest were designed using the Merck online tool and synthesized by Sangon Biotech (China). The shRNA oligonucleotides were cloned into the pLKO.1 vector (#10878, Addgene, USA) using T4 DNA ligase (M0202T, NEB, USA). The ligation product was transformed into DH5α competent cells (11803ES80, YEASEN, China), and positive colonies were screened and sequenced to confirm correct insertion.

For lentivirus production, the pLKO.1-shRNA plasmid was co-transfected with psPAX2 (#12260, Addgene, USA) and PMD2.G (#12259, Addgene, USA) into HEK-293T cells (CL-000, PriCella, China) using standard calcium phosphate or lipid-based transfection protocols. Lentiviral supernatants were collected at 24- and 48-hours post-transfection, centrifuged, and filtered for use in subsequent infection steps.

Target ESCC cells were infected with the filtered Lenti-shRNA-containing supernatant for 48 hours. Following infection, cells were selected in complete medium containing 2 μg/mL puromycin (ST551, Beyotime, China) to enrich for transduced populations. Puromycin-resistant cells were then expanded in medium with 1 μg/mL puromycin until stable cell lines were established 16. All shRNA sequences used are listed in Table S1.

### 2.4 Tumor-associated PCR array analysis

Comprehensive gene expression analysis was performed using the WC-MRNA0188-H PCR array platform (Wc Gene Biotech Co., Ltd., Shanghai, China). All procedures were carried out in accordance with the manufacturer's instructions to ensure consistency and reproducibility across all biological replicates.

### 2.5 Clinical sample collection

A total of 33 pairs of ESCC tissues and matched adjacent nontumor esophageal tissues were obtained from Gansu Provincial People's Hospital. Following surgical resection, all tissue samples were immediately snap-frozen in liquid nitrogen for subsequent molecular analyses. Sample collection and processing were conducted in accordance with informed consent regulations and approved institutional ethical guidelines.

### 2.6 Transwell assay

For migration and invasion assays, cells were suspended at a concentration of 2 × 10^5^ cells/mL in 200 μL RPMI-1640 medium supplemented with 1% FBS, and seeded into the upper chambers of Transwell inserts. The lower chambers were filled with 800 μL RPMI-1640 medium containing 10% FBS to serve as a chemoattractant. After 24 hours of incubation, non-migrated cells on the upper membrane surface were gently removed with a cotton swab. Migrated or invaded cells on the lower surface were fixed in methanol and stained with crystal violet. For invasion assays, the upper chambers were pre-coated with Matrigel (Corning, USA), whereas migration assays were performed without Matrigel.

### 2.7 Xenograft experiments

Female immunodeficient BALB/c nude mice (4-5 weeks old) were obtained from an accredited vendor (Lanzhou Veterinary Research Institute, CAAS) and acclimatized in temperature- and humidity-controlled SPF facilities.

EC109 cells (5×10^7^) were either untreated or pretreated (rhCCL15), then digested with trypsin, washed with PBS, and centrifuged. The collected cells were resuspended in PBS and subcutaneously injected into the mid-posterior axillary region of the mice to establish tumors.

Animals were randomly allocated into four experimental cohorts (n=5) as follows: (1) Control group (injected with untreated EC109 cells), (2) rhCCL15 group (injected with EC109 cells pretreated with rhCCL15), (3) shRNA-CCR1 group (injected with EC109 cells with CCR1 knockdown) and (4) rhCCL15 + shRNA-CCR1 group (injected with CCR1-knockdown EC109 cells pretreated with rhCCL15).

At the endpoint, mice were euthanized using isoflurane inhalation (YaJi Biological, Shanghai, China). Tumors were excised, weighed, and either snap-frozen at -80°C or fixed in 4% paraformaldehyde (PFA) for further analyses.

### 2.8 RT-qPCR analysis

Total RNA was extracted from tumor tissues or EC109/TE-1 cells using TRIzol (Invitrogen, USA). Following quantification, 1 μg of RNA was reverse transcribed into cDNA (Takara, Japan). Quantitative PCR (qPCR) was performed using diluted cDNA, gene-specific primers, and SYBR Green Master Mix (Vazyme, China). Relative mRNA expression levels were calculated using the 2^^-ΔΔCt^ method 17. The primer sequences used are provided in Table S2.

### 2.9 Western Blotting Analysis

Total protein was extracted from EC109 and TE-1 cells using RIPA lysis buffer (Beyotime, China) supplemented with protease and phosphatase inhibitors. Lysates were incubated on ice for 30 minutes and then centrifuged at 12,000 rpm for 15 minutes at 4°C. Protein concentrations were determined using the BCA protein assay kit. Equal amounts of protein (20 μg) were resolved on 10% SDS-PAGE gels and transferred to PVDF membranes. After blocking with 5% BSA for 2 hours at room temperature, the membranes were incubated overnight at 4°C with primary antibodies against CCR1, p-AKT, total AKT, p-ERK1/2, total ERK1/2, and GAPDH. After washing, membranes were incubated with HRP-conjugated secondary antibodies for 1 hour at room temperature. Protein bands were visualized by chemiluminescence, and band intensities were quantified relative to GAPDH. Details of the antibodies used are listed in Table S3.

### 2.10 Transcription factor prediction

The 1000 bp upstream DNA sequence from the transcription start site (TSS) of the CDK2 gene was submitted to Cistrome DB (*http://cistrome.org/db/#/*) to transcription factors prediction. The transcription factors were ranked in descending order based on their predicted regulatory potential.

### 2.11 Data statistics and analysis

Statistical analyses were conducted using GraphPad Prism or SPSS software. One-way ANOVA was used to compare multiple independent groups, while two-tailed unpaired Student's t-tests were employed for comparisons between two groups. Data are presented as mean ± standard error of the mean (SEM). A p-value of <0.05 was considered statistically significant.

## 3. Results

### 3.1 CCR1 and CCL15 are significantly upregulated in ESCC cells

**Figure 1:**
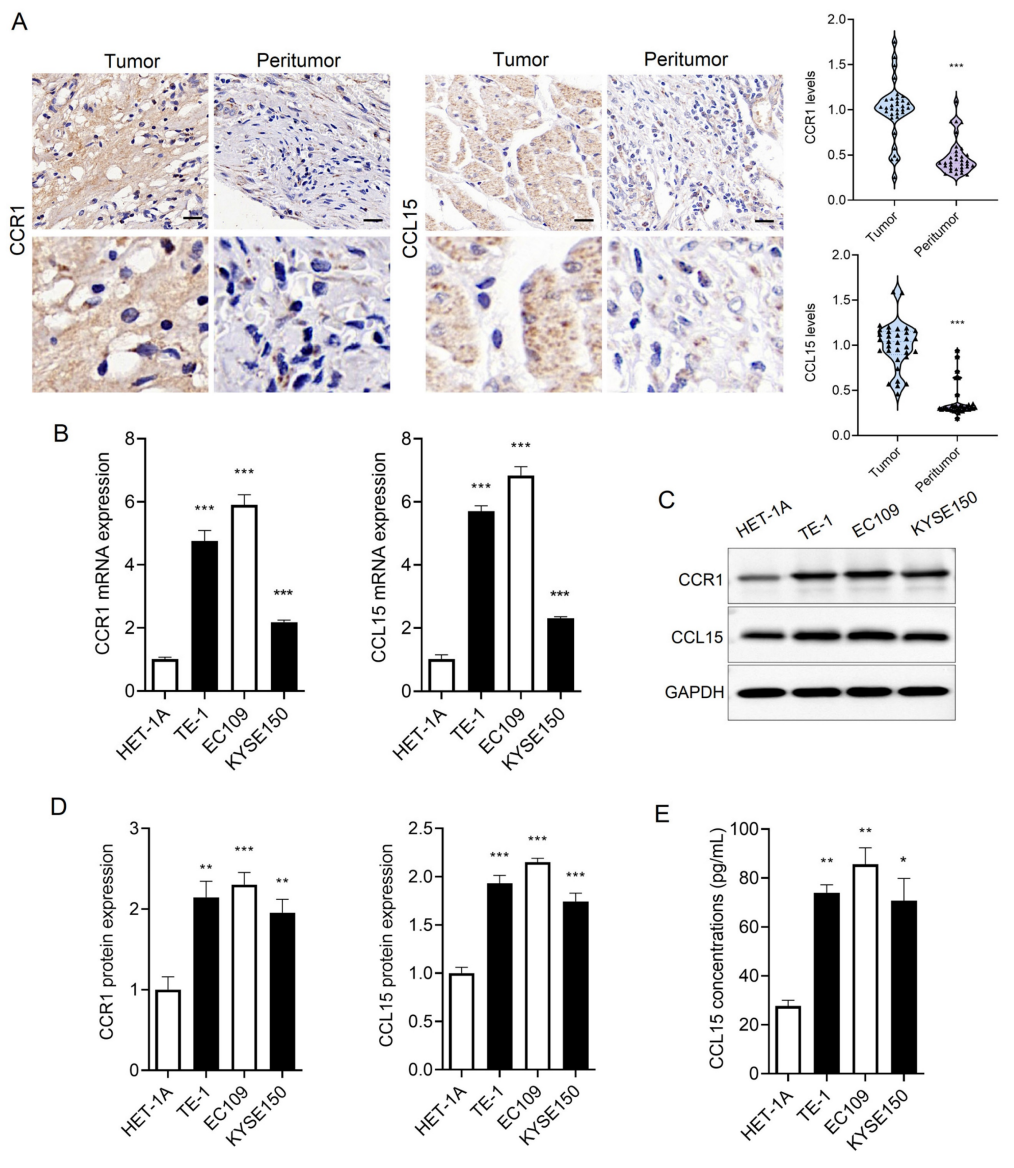
CCR1 and CCL15 are highly expressed in ESCC. (A) Immunohistochemical results and statistics of CCR1 and CCL15. (B) Statistics of mRNA levels of CCR1 and CCL15 in HET-1A, TE-1, EC109 and KYSE150 cells. (C-D) Western blotting analysis of CCR1 and CCL15 protein levels in HET-1A, TE-1, EC109 and KYSE150 cells. (E) ELISA analysis of CCL15 in cell culture medium supernatants. * vs. Tumor/HET-1A, *p<0.05, **p<0.01, ***p<0.001.

We collected ESCC tissue and matched adjacent non-tumor tissues from clinical patients. Both CCR1 and CCL15 were markedly overexpressed in ESCC tissues compared to adjacent non-tumor tissues (Figure 1A). Furthermore, we examined CCR1 and CCL15 expression levels in esophageal epithelial cells (HET-1A) and ESCC cells (TE-1, EC109, and KYSE150) using RT-qPCR and WB. The basal expression of CCR1 and CCL15 were significantly elevated in TE-1, EC109, and KYSE150 cells compared to HET-1A cells (Figure 1B-1D). As a secreted chemokine, CCL15 levels in cell culture supernatants were also quantified using ELISA. CCL15 concentrations were significantly higher in the supernatants of TE-1, EC109, and KYSE150 cells compared to HET-1A cells (Figure 1E). These findings suggest that CCR1 and CCL15 may serve as important contributors to ESCC progression.

### 3.2 CCL15 promotes ESCC cell proliferation and migration

**Figure 2:**
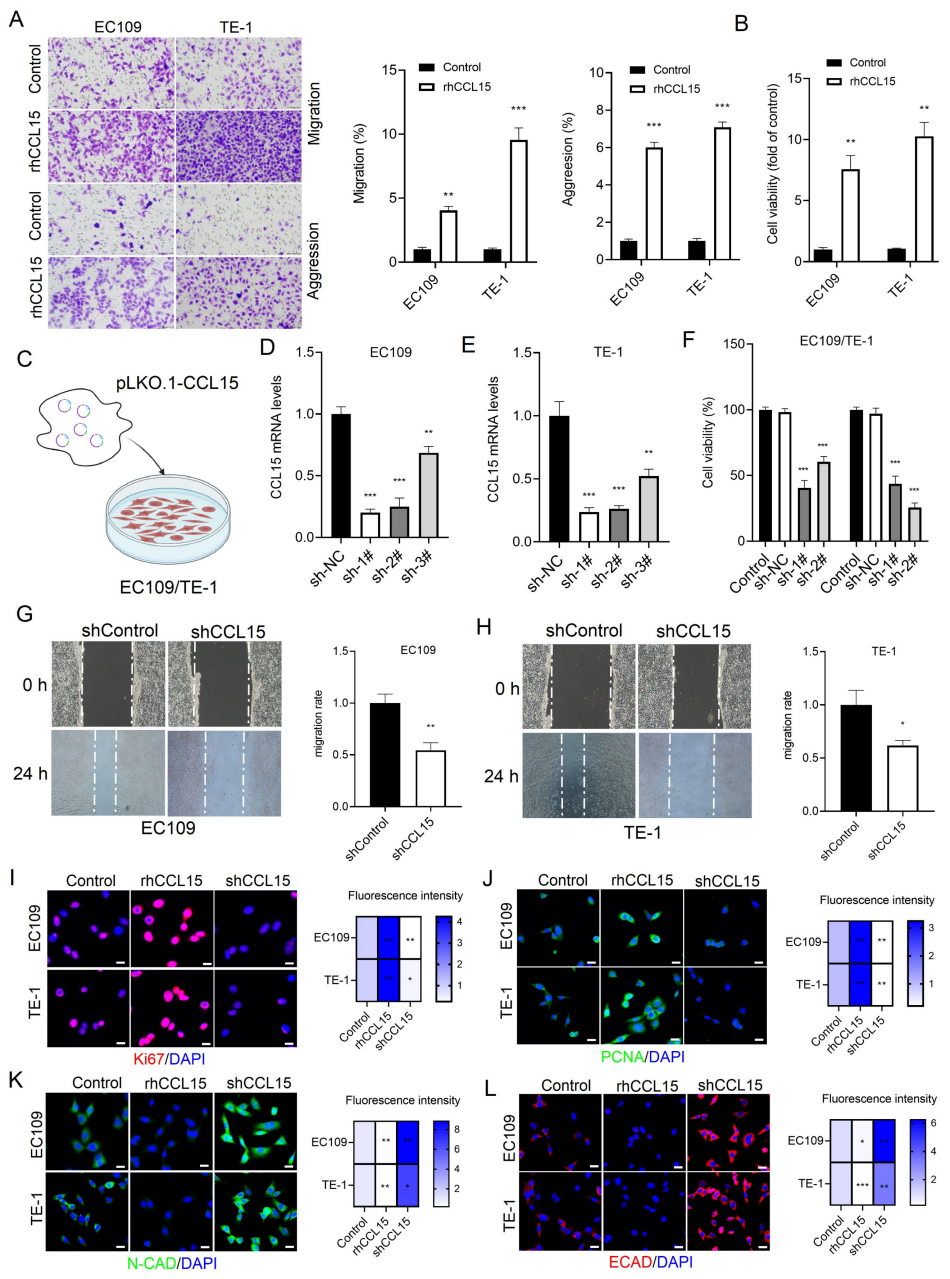
CCL15 mediates ESCC proliferation and migration. (A). Crystal violet staining shows changes in cell migration and invasion. (B) Cell viability was analyzed by CCK8. (C) Schematic diagram of lentivirus-infected EC109 and TE-1 cells. (D-E) RT-qPCR for analyzing mRNA levels of CCL15. (F) Cell viability was analyzed by CCK8. (G-H) Cell scratch assay for analyzing cell migration. (I-L) Immunofluorescence analysis of Ki67, PCNA, E-CAD and N-CAD protein levels in EC109 and TE-1 cells. * vs. Control/shNC/shControl, *p<0.05, **p<0.01, ***p<0.001.

To explore the functional role of CCL15 in ESCC, EC109 and TE-1 cells were treated with rhCCL15 *in vitro*. Transwell assays revealed that rhCCL15 significantly enhanced the migration and invasion capabilities of ESCC cells (Figure 2A). The effect of CCL15 on cell proliferation was assessed using the CCK-8 assay. We observed rhCCL15 treatment significantly promoted the proliferation of both EC109 and TE-1 cells (Figure 2B).

To further investigate the role of CCL15, three shRNA sequences targeting CCL15 were designed and packaged into lentiviral vectors (lenti-shCCL15). Following infection of ESCC cells (Figure 2C-2E), cell proliferation assays showed that CCL15 knockdown significantly inhibited cell proliferation (Figure 2F). Wound-healing assays were performed to assess cell migration, and the results demonstrated that CCL15 silencing markedly reduced the migratory capacity of ESCC cells (Figure 2G-2H).

Ki67 and PCNA are established markers of cell proliferation and widely used to evaluate tumor cell growth 18. Immunofluorescence analysis showed that rhCCL15 treatment increased the percentage of Ki67- and PCNA-positive cells, whereas CCL15 knockdown significantly reduced their expression (Figure 2I-2J). E-cadherin (E-CAD) and N-cadherin (N-CAD), critical adhesion molecules involved in epithelial-mesenchymal transition (EMT), were also assessed. rhCCL15 treatment significantly suppressed the expression of E-CAD and N-CAD, while CCL15 knockdown restored their levels (Figure 2K-2L).

### 3.3 CCL15 promotes ESCC progression by CCR1

**Figure 3:**
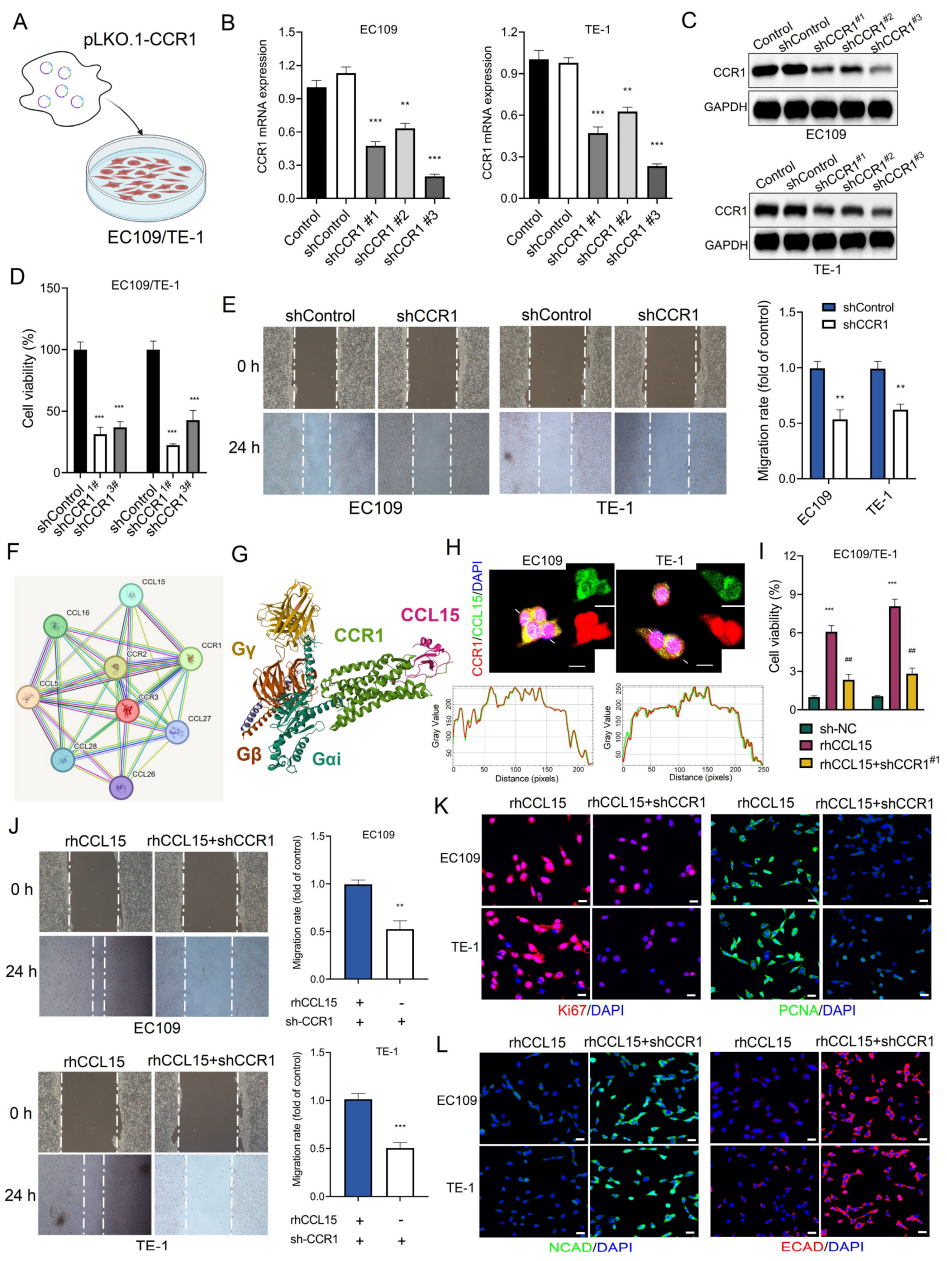
CCL15 promotes ESCC proliferation and migration through CCR1. (A) Schematic diagram of lentivirus-infected EC109 and TE-1 cells. (B) RT-qPCR for analyzing mRNA levels of CCR1. (C) Western blotting analysis of protein level changes in CCR1. (D) Cell viability was analyzed by CCK8. (E) Cell migration analysis of EC109 and TE-1. (F) Protein-protein interaction (PPI) analysis. (G) PBD (7VLA) demonstrates the binding pattern of CCR1 and CCL15. (H) Immunofluorescence co-localization analysis of CCR1 and CCL15. Image J software was used for co-localization analysis. (I) Cell viability was analyzed by CCK8. (J) Cell migration analysis of EC109 and TE-1. (K-L) Immunofluorescence analysis of Ki67, PCNA, E-CAD and N-CAD protein levels in EC109 and TE-1 cells. * vs. Control/shControl, *p<0.05, **p<0.01, ***p<0.001; ^#^ vs. rhCCL15, ^###^*p*<0.001.

Although previous studies on the CCL15-CCR1 axis have predominantly focused on tumor-derived CCL15 recruiting CCR1⁺ immune cells, our findings revealed that CCR1 is expressed at significantly higher basal levels in ESCC cells compared to normal esophageal epithelial HET-1A cells. This observation led us to hypothesize that tumor-intrinsic CCR1 may serve as a functional receptor activated by CCL15 to promote tumor progression. To test this hypothesis, we cloned shRNA sequences targeting CCR1 into the pLKO.1 vector and co-transfected them with psPAX2 and pMD.2G into 293T cells to generate lentiviral particles (lenti-shCCR1). After infecting EC109 and TE-1 cells with lenti-shCCR1, RT-qPCR analysis confirmed effective silencing of CCR1 mRNA by all three shRNA constructs (Figure 3A-3B). Western blotting further validated the knockdown of CCR1 protein expression (Figure 3C). Functionally, CCK-8 and wound-healing assays showed that CCR1 knockdown significantly suppressed cell proliferation and migration (Figure 3D-3E).

Using the PPI database, we identified a direct interaction between CCL15 and CCR1 (Figure 3F). Structural analysis *via* the PDB database revealed that CCL15 binds to the extracellular domain of CCR1 (Figure 3G). Immunofluorescence co-localization and co-immunoprecipitation analyses confirmed the physical interaction between CCL15 and CCR1 in ESCC cells (Figure 3H and S2A-S2B). Notably, CCR1 knockdown reversed rhCCL15-induced increase in cell proliferation (Figure 3I) and attenuated the upregulation of Ki67 and PCNA expression induced by rhCCL15 (Figure 3K). Similarly, wound-healing assays demonstrated that CCR1 silencing reversed the enhanced migratory phenotype caused by rhCCL15 (Figure 3J). Additionally, CCR1 knockdown restored E-CAD and N-CAD expressions, which were otherwise downregulated upon rhCCL15 treatment (Figure 3L).

**Figure 4:**
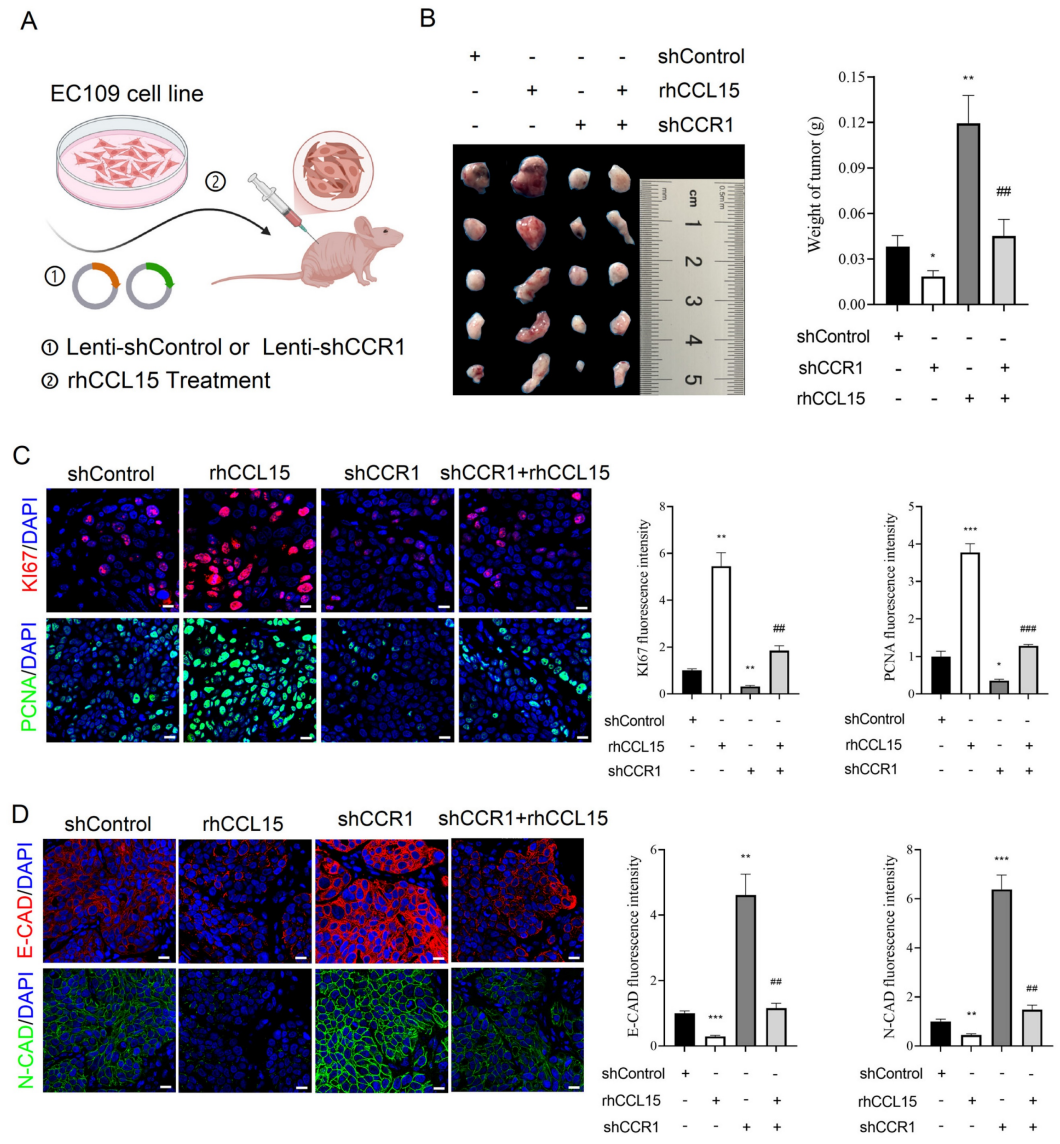
CCL15 promotes ESCC progression *via* CCR1. (A) Flow chart of xenograft experiment. Subcutaneous injection of treated or untreated EC109 cells into nude mice. (B) Statistical analysis of tumor quality. (C) Immunofluorescence analysis of Ki67 and PCNA levels in tumor tissues. (D) Immunofluorescence for analysis of E-CAD and N-CAD levels in tumor tissues. * vs. shControl, *p<0.05, **p<0.01, ***p<0.001; ^#^ vs. rhCCL15, ^##^*p*<0.01, ^###^*p*<0.01.

To further validate the in vivo relevance of the CCL15-CCR1 axis in ESCC progression, we conducted a subcutaneous xenograft model in nude mice. Specifically, EC109 cells were allocated to four cohorts: Control, rhCCL15-treated, rhCCL15-treated + CCR1 knockdown, and CCR1 knockdown. Cells were injected into the mid-axillary subcutaneous region of the mice (Figure 4A). Tumors derived from rhCCL15-treated EC109 cells were larger than the control, while CCR1 knockdown led to marked tumor growth inhibition (Figure 4B). Notably, CCR1 knockdown reversed the tumor-promoting effect of rhCCL15 (Figure 4B). We further assessed cell proliferation by examining Ki67 and PCNA expression, and evaluated cell migration through E-CAD and N-CAD levels in tumor tissues. Consistent with the *in vitro* findings, rhCCL15 treatment significantly increased the number of Ki67- and PCNA-positive cells (Figure 4C), while reducing the expression levels of E-CAD and N-CAD (Figure 4D). These effects were reversed by CCR1 knockdown (Figure 4C-4D). Collectively, these results demonstrate that CCL15 promotes ESCC progression by acting on tumor-intrinsic CCR1.

### 3.4 CDK2 mediates CCL15-CCR1-induced proliferation and migration in ESCC

**Figure 5:**
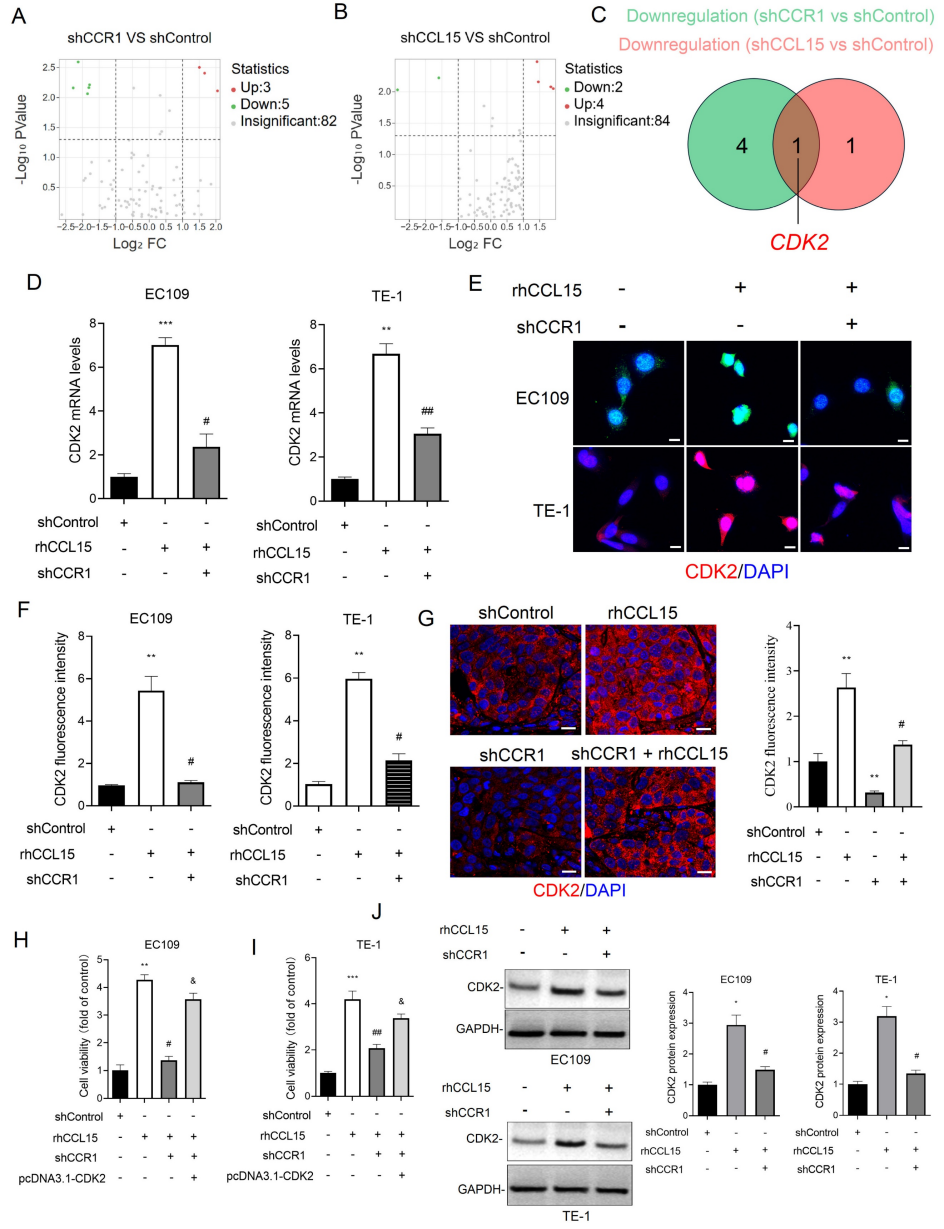
CCL15 promotes ESCC cell proliferation *via* CCR1-mediated CDK2 transcriptional activation. (A-B) PCR array analysis of tumor-related gene changes in EC109 cells after CCR1 and CCL15 knockdown. (C) Venn diagram showing downregulated genes. (D) RT-qPCR for analyzing mRNA levels of CDK2. (E-G) Immunofluorescence for analysis of CDK2 levels in EC109, TE-1 cells and tumor tissues. (H-I) Cell viability was analyzed by CCK8 assay. (J) Western blotting for analyzing protein levels of CDK2. * vs. shControl, **p<0.01, ***p<0.001; ^#^ vs. rhCCL15, ^#^ *p*<0.05, ^# #^*p*<0.01; ^&^ vs. shCCR1 + rhCCL15,^ &^*p*<0.01.

To investigate the mechanism by which CCL15-CCR1 axis promotes ESCC progression, we conducted a tumor-related gene PCR array analysis in EC109 cells following CCL15 and CCR1 knockdown. Notably, CDK2 expression was significant after silencing either CCL15 or CCR1 (Figure 5A-5C). *In vitro*, rhCCL15 treatment elevated CDK2 mRNA levels in both EC109 and TE-1, while CCR1 knockdown effectively reversed this upregulation (Figure 5D). The protein expression of CDK2 was consistent with the mRNA trends observed (Figure 5E-5F and 5J). Similarly, *in vivo* analysis showed that rhCCL15 treatment increased CDK2 expression at both mRNA and protein levels, whereas CCR1 knockdown reversed these effects (Figure 5G). These findings support that CCL15-CCR1 axis positively regulates the transcriptional activation of CDK2 in ESCC. As a core regulator of cell cycle, CDK2 becomes activated during the late G1 phase and remains active throughout the S phase, playing a critical role in cancer cell proliferation 19. Based on this, we hypothesized that CDK2 mediates the CCL15-CCR1 axis-induced proliferation and migration of ESCC cells. We cloned the CDS of *Cdk2* into the pcDNA3.1 vector and overexpressed *Cdk2* in cells treated with rhCCL15 and subjected to CCR1 knockdown. CCK-8 assay showed that CDK2 overexpression weakened the suppressive effect of CCR1 knockdown (Figure 5H-5I).

### 3.5 CCL15-CCR1 activates CDK2 transcription through AKT and ERK1/2

**Figure 6:**
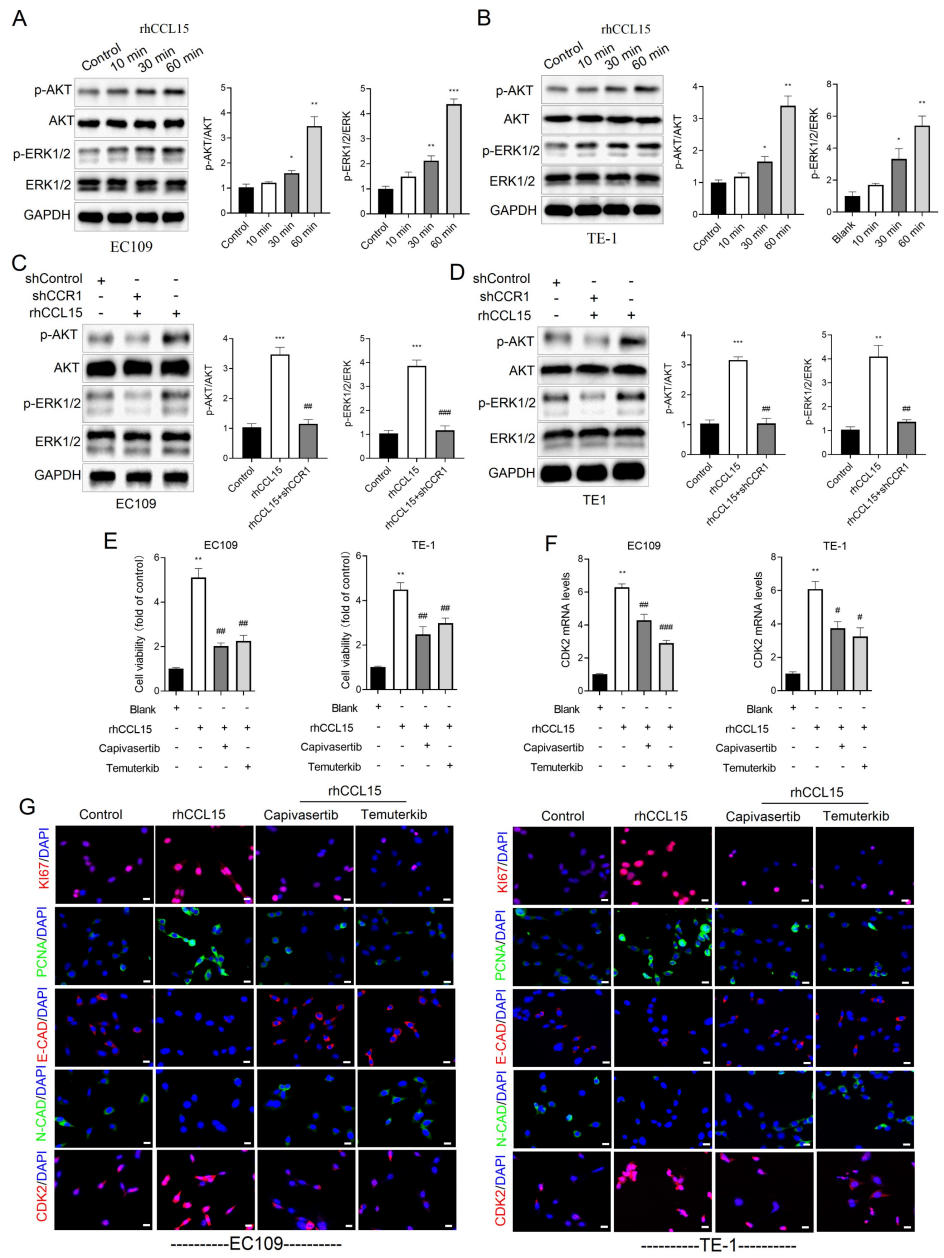
CCL15-CCR1 activates CDK2 transcription *via* AKT and ERK1/2 in ESCC. (A-D) Western blotting analysis of protein level changes in p-AKT, AKT, ERK1/2, p-ERK1/2 and GAPDH. (E) Cell proliferation of EC109 and TE-1 cells detected using CCK8. (F) Analysis of CDK2 mRNA levels in EC109 and TE-1 cells by RT-qPCR. (G) Immunofluorescence analysis of Ki67, PCNA, E-CAD, N-CAD and CDK2. * vs. Control/Blank, *p<0.05, **p<0.01, ***p<0.001; ^#^ vs. rhCCL15, ^#^*p*<0.05, ^##^*p*<0.01, ^###^*p*<0.01.

In ESCC, the PI3K/AKT and ERK1/2 signaling are known to regulate cell proliferation, apoptosis, and migration 20. As a G-protein coupled receptor, CCR1 can activate multiple downstream signaling cascades, including PI3K-AKT and ERK1/2 12, 13. To investigate whether the CCL15-CCR1 axis regulates CDK2 transcription through these pathways, we performed a series of *in vitro* experiments. Our experiments demonstrated that rhCCL15 treatment increased the phosphorylation of AKT (p-AKT) and ERK1/2 (p-ERK) in ESCC cells (Figure 6A-6B). Notably, CCR1 knockdown abolished the rhCCL15-induced activation of both p-AKT and p-ERK (Figure 6C-6D), indicating that rhCCL15 activates AKT and ERK1/2 signaling through CCR1. To further confirm this, we treated cells with Temuterkib (an ERK1/2 inhibitor) and Capivasertib (an AKT inhibitor). Both inhibitors significantly reduced the rhCCL15-induced upregulation of CDK2 at both the mRNA and protein levels (Figure 6F-6G). Additionally, CCK-8 assays showed that Temuterkib and Capivasertib reversed the rhCCL15-induced proliferation of ESCC cells (Figure 6E). Immunofluorescence analysis further confirmed that these inhibitors attenuated the increase in Ki67- and PCNA-positive cells induced by rhCCL15 (Figure 6G). Also, these inhibitors restored E-CAD and N-CAD levels, which were otherwise downregulated by rhCCL15 (Figure 6G). These findings demonstrate that CCL15-CCR1 axis promotes CDK2 transcription and ESCC progression through activation of the AKT and ERK1/2 pathways.

### 3.6 AKT and ERK1/2 phosphorylate c-Jun to mediate CDK2 transcription activation

**Figure 7:**
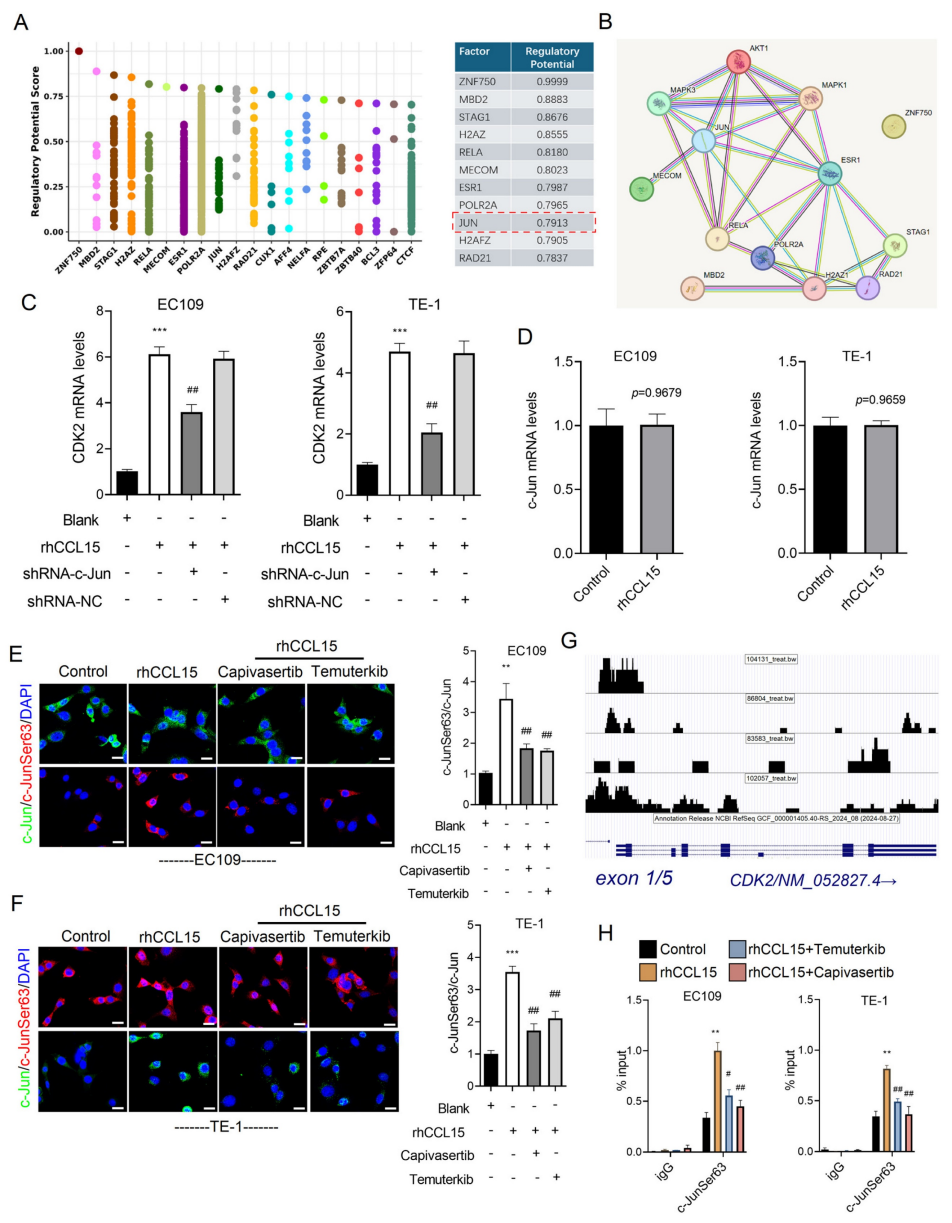
AKT and ERK1/2 mediate c-Jun phosphorylation and initiate CDK2 transcription. (A) Cistrome DB demonstrates transcription factors that regulate CDK2 transcription. (B) PPI analysis of transcription factors and AKT and ERK1/2. (C) Analysis of CDK2 mRNA levels in EC109 and TE-1 cells by RT-qPCR. (D) Alteration of c-Jun mRNA levels in EC109 and TE-1 cells. (E-F) Immunofluorescence analysis of c-Jun and p-c-Jun (Ser63). (G) The UCSC Genome Browser indicates p-c-Jun (Ser63) binding to the CDK2 promoter region. (H) ChIP-qPCR analysis of p-c-Jun binding to the CDK2 promoter in EC109 and TE-1 cells. * vs. Blank/Control, **p<0.01, ***p<0.001; ^#^ vs. rhCCL15, ^##^*p*<0.01, ^###^*p*<0.01.

To further explore the downstream mechanism by which AKT and ERK1/2 regulate CDK2 transcription, we analyzed the CDK2 promoter region using the Cistrome DB database. Specifically, we submitted a 1000 bp sequence upstream of the transcription start site (TSS) and identified 14 transcription factors with a prediction score greater than 0.78, including ZNF750, MBD2, STAG1, H2AZ, RELA, MECOM, ESR1, POLR2A, JUN (c-Jun), H2AFZ, and RAD21 (Figure 7A). PPI network analysis further revealed that c-Jun not only regulates CDK2 but also interacts with AKT and ERK1/2 (Figure 7B). Based on this observation, we hypothesized that AKT and ERK1/2 modulate CDK2 transcription via phosphorylation of c-Jun. Supporting this hypothesis, c-Jun knockdown significantly suppressed CDK2 mRNA expression in rhCCL15-treated EC109 and TE-1 cells (Figure 7C). Interestingly, rhCCL15 treatment did not alter total c-Jun mRNA levels (Figure 7D), suggesting a post-transcriptional mechanism. Immunofluorescence analysis confirmed that while total c-Jun protein levels remained unchanged following rhCCL15 treatment, the phosphorylation of c-Jun at Ser63 (p-c-Jun) was markedly increased in both cell lines (Figure 7E-7F). Treatment with Temuterkib and Capivasertib inhibited this rhCCL15-induced phosphorylation of c-Jun (Figure 7E-7F). Furthermore, the rhCCL15 treatment enhanced the binding of p-c-Jun to the CDK2 promoter, an effect that was abolished by both AKT and ERK1/2 inhibitors (Figure 7G-7H). Taken together, these findings indicate that in ESCC cells, the CCL15-CCR1 axis promotes CDK2 transcriptional activation through AKT- and ERK1/2-mediated phosphorylation of c-Jun at Ser63.

### 3.7 Screening of a natural organic compound library identifies Jervine as a CCR1 inhibitor

**Figure 8:**
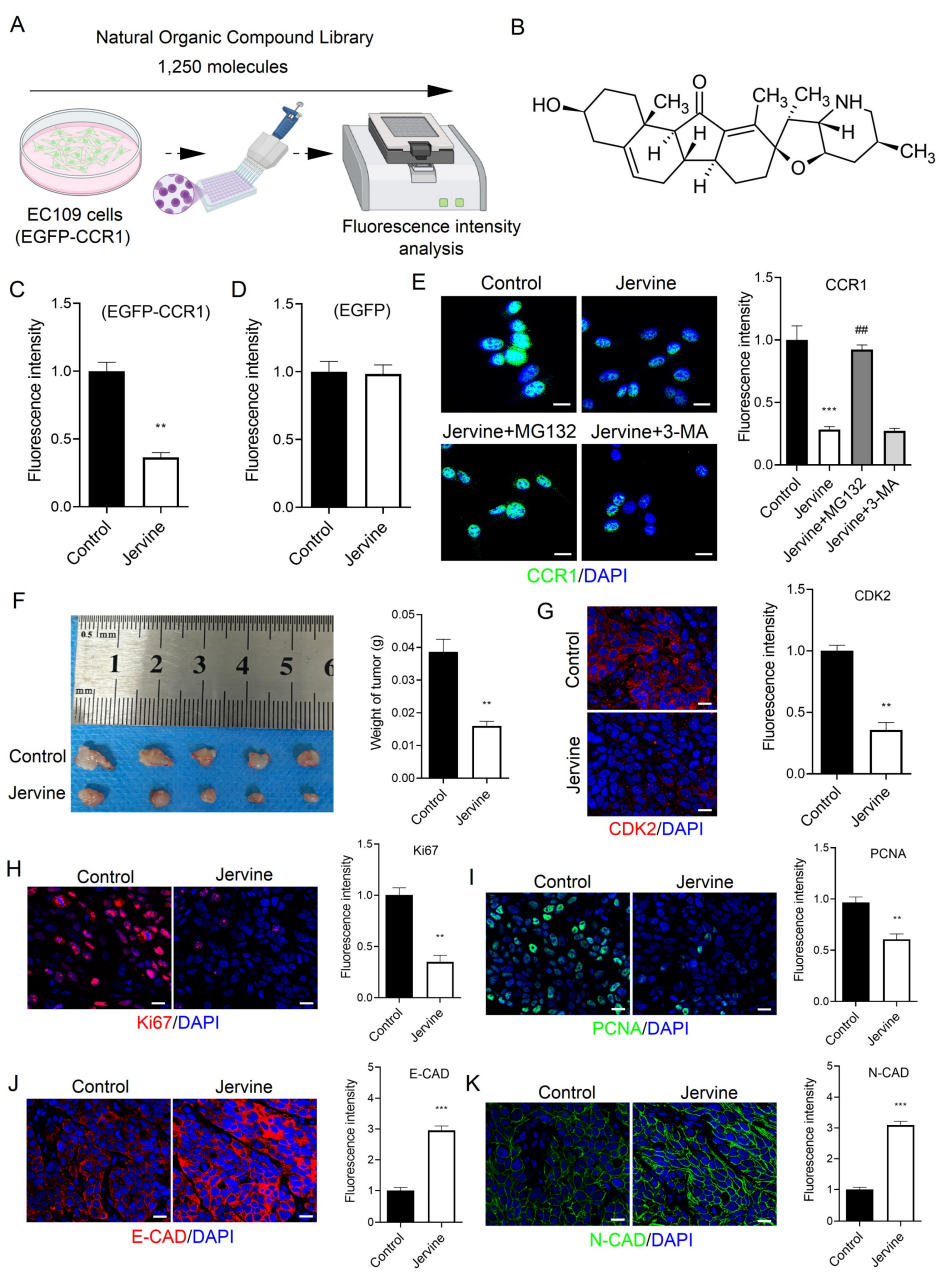
Jervine promotes CCR1 degradation and treats ESCC. (A) Natural organic drug library screening process for CCR1 inhibitors. (B) Molecular structure of Jervine. (C-D) Fluorescence intensity analysis of EGFP-CCR1 and control EGFP constructs. (E) Immunofluorescence for analyzing changes in CCR1 levels. (F) Statistical analysis of tumor quality. (G-K) Immunofluorescence analysis of CDK2, Ki67, PCNA, E-CAD and N-CAD levels in tumor tissues. * vs. Control, **p<0.01, ***p<0.001. ^#^ vs. Jervine group, ^##^*p*<0.01.

Targeted drug screening is a critical strategy in cancer therapy. In this study, we screened a library of 1,250 plant-derived natural organic compounds to identify potential CCR1 inhibitors in ESCC cells (Figure 8A). EC109 cells stably expressing EGFP-tagged CCR1 were treated with each compound (n=2) at a concentration of 10 μM for 72 hours. Among the candidates, three compounds-Troxerutin, Guaiacol, and Jervine-significantly reduced CCR1 levels (Figure S1A-S1C). A secondary screen (n=6) identified Jervine consistently and robustly downregulated CCR1 expression (Figure 8B-8C). To rule out the possibility that the reduction in fluorescence was due to a decrease in EGFP protein rather than CCR1-specific degradation, we treated EC109 cells expressing EGFP alone with Jervine. The fluorescence intensity remained unchanged (Figure 8D), indicating that Jervine specifically decreases CCR1 protein levels rather than EGFP fluorescence.

Since protein degradation can occur *via* the ubiquitin-proteasome or autophagy-lysosome pathways 21, we investigated the underlying mechanism by treating cells with the proteasome inhibitor MG132 and the autophagy inhibitor 3-MA. Notably, MG132, but not 3-MA, reversed the CCR1 degradation induced by Jervine (Figure 8E), suggesting that Jervine promotes CCR1 degradation via the ubiquitin-proteasome pathway, not the autophagy-lysosome pathway. We further evaluated the therapeutic potential of Jervine *in vivo* using a subcutaneous xenograft model. EC109 cells, either untreated or pretreated with 10 μM Jervine, were injected subcutaneously into nude mice. Jervine treatment significantly inhibited tumor growth (Figure 8F), reduced Ki67 and PCNA positive cell numbers (Figure 8H-8I and S3A-S3B) and restored E-CAD and N-CAD expression (Figure 8J-8K). Additionally, Jervine significantly inhibited the transcription of CDK2 (Figure 8G and S3A-S3B). Together, these findings indicate that Jervine is a natural compound that selectively targets CCR1, promotes its degradation via the proteasome pathway, and effectively suppresses ESCC progression.

## 4. Discussion

Due to its late-stage diagnosis, high metastatic potential, and resistance to chemotherapy, ESCC is regarded as one of the most aggressive cancers and lethal malignancies 22-24. Although significant challenges remain in treating ESCC, encouragingly, a growing number of therapeutic targets have been identified in recent years 25-29. Among them, the chemokine family, including CC (e.g., CCL1, CCL3, CCL5, CCL7, and CCL15), CXC (e.g., CXCL1, CXCL3, CXCL5, and CXCL10), XC (e.g., XCL1 and XCL2), and CX3C (e.g., CX3CL1), plays a pivotal role in tumor development, metastasis, and chemotherapy resistance 30-33. Among these chemokines, CCL15 has been found to be highly expressed in a variety of tumor tissues. Yamamoto *et al.* revealed that CCL15 contributes to pulmonary metastasis in rectal cancer 8, and Liu *et al.* found that CCL15 facilitates immune evasion in hepatocellular carcinoma by regulating immune-related signaling pathways 4. Furthermore, CCL15 has been identified as both a biomarker and tumor promoter in gastric cancer 34, melanoma 35, and leukemia 36. However, the function of CCL15 on ESCC has not been well characterized. In our study, we observed that CCL15 expression was significantly elevated in ESCC tumor tissues compared to adjacent nontumor tissues, and similarly, ESCC cell lines exhibited higher CCL15 levels than normal esophageal epithelial cells. These findings suggest that CCL15 may be involved in promoting ESCC progression. To investigate this, we performed gain- and loss-of-function experiments using rhCCL15 treatment and CCL15 knockdown. Consistent with our hypothesis, CCL15 indeed promoted ESCC cell proliferation and migration. Additionally, subcutaneous xenograft experiments in nude mice verified that CCL15 promotes tumor growth *in vivo*.

CCL15 can activate multiple receptors, including CCR3 and CCR1 37. Among these, CCR1 is considered the primary receptor mediating the tumor-promoting effects of CCL15, and it has been implicated in cancer metastasis, drug resistance, and progression. Notably, most studies to date have focused on the paracrine function of CCL15—namely, its role in recruiting CCR1⁺ immune cells to the tumor microenvironment. For instance, in hepatocellular carcinoma, CCL15 recruits CCR1⁺ monocytes to support immune evasion and metastasis 38. Similarly, in colorectal cancer, THRC1-induced upregulation of CCL15 facilitates the recruitment of CCR1⁺ macrophages, thereby enhancing tumor progression 39. In contrast, Yamamoto *et al.* found that CCR1-positive cells recruited by CCL15 were mainly tumor-associated neutrophils (CD11b^+^, CD33^-^, HLA-DR^-^, CD15^+^ and CD16^+^), with a small proportion being granulocytic myeloid-derived suppressor cells 8. These cells contribute to the formation of an immunosuppressive tumor microenvironment and promote tumor progression by producing MMP9 and ARG1. However, less attention has been paid to the tumor-intrinsic role of CCR1. In our study, we found that CCR1 protein levels were elevated not only in ESCC tissues but also in ESCC cell lines, compared to adjacent nontumor tissues and normal epithelial cells. This indicates that CCR1 may contribute to ESCC progression and could be activated by CCL15 in ESCC cells. To test this, we used lentiviral knockdown of CCR1 and confirmed that it's silencing significantly suppressed ESCC cell proliferation and migration *in vitro*. Furthermore, subcutaneous xenograft assays in nude mice demonstrated that CCR1 promotes tumor growth *in vivo*. Importantly, CCR1 knockdown reversed the tumor-promoting effects of CCL15, providing strong evidence that CCL15 exerts its oncogenic role in ESCC primarily through CCR1 activation. These findings expand our understanding of the CCL15-CCR1 axis, revealing a previously underappreciated tumor-intrinsic mechanism contributing to ESCC progression.

Next, we explored the potential molecular mechanisms through which the CCL15-CCR1 axis promotes ESCC proliferation and migration. PCR array analysis showed that *Cdk2* expression was downregulated in EC109 cells following either CCR1 or CCL15 knockdown. CDK2 contributes to mediate cell cycle, and its aberrant activation has been implicated in the pathogenesis of various cancers, such as pulmonary 19, mammary gland 40, hepatic 41, and esophageal squamous epithelium 42 carcinogenesis. Lei *et al.* demonstrated that autophagy deficiency mediated by ARRB1 blocks the cell cycle and promotes hepatocellular carcinoma progression via the CDK2-CCNE1 complex 41. Hu *et al.* reported that CDK2 promotes tumorigenesis in lung cancer by regulating centriolar protein CP110, particularly in KRAS-mutant cases 19. In ESCC, CDK2 has been shown to drive malignant progression through the Rb/E2F2/RRM2 signaling pathway 42. Given its central role in tumor biology, CDK2 is regarded as a promising therapeutic target, and its targeted degradation is considered an effective treatment strategy 43-45. Based on these findings, we hypothesize that CDK2 may be involved in mediating the effects of CCL15-CCR1 axis in ESCC. To test this, we treated ESCC cells with rhCCL15, silenced CCR1, and simultaneously overexpressed CDK2. The results confirmed that CDK2 mediates the CCL15-CCR1-induced enhancement of proliferation and migration in ESCC cells.

Phosphorylation of AKT and ERK1/2 are key downstream molecular events mediating CCR1 signaling. CCR1 can activate the AKT-ESR1 to promote hepatocellular carcinoma cell proliferation and migration 46 and enhances prostate cancer cell invasive via activating ERK1/2 and Rac signaling 14. In our study, rhCCL15 treatment enhanced AKT and ERK1/2 phosphorylation, whereas CCR1 knockdown abrogated this effect in ESCC cells. To assess the functional relevance of these pathways, we treated ESCC cells with Temuterkib (ERK1/2 inhibitor) and Capivasertib (AKT inhibitor). Both treatments significantly reduced CDK2 transcription, suggesting that AKT and ERK1/2 mediate the transcriptional activation of CDK2 downstream of CCL15-CCR1. Next, we investigated the transcriptional regulators of CDK2 and identified c-Jun as a candidate transcription factor with high regulatory potential. Notably, AKT and ERK1/2 can phosphorylate c-Jun, modulating its transcriptional activity 47. These findings suggest that CCL15-CCR1-induced transcriptional activation of CDK2 may be mediated by c-Jun phosphorylation *via* AKT and ERK1/2. Using lentiviral knockdown of c-Jun, we confirmed that c-Jun is essential for AKT/ERK1/2-mediated upregulation of CDK2. The tryptophan at position 63 of c-Jun is the main phosphorylation site and plays a key role in c-Jun's transcriptional activation 48. In our study, pharmacological inhibition of AKT and ERK1/2 abolished CCL15-induced c-Jun phosphorylation and markedly decreased the binding of p-c-Jun to the CDK2 promoter. In summary, these results suggest that in ESCC, the transcriptional activation of CDK2 by CCL15-CCR1 is mediated by c-Jun phosphorylation through AKT as well as ERK1/2.

Inhibitors targeting CCR1—such as BX471, J113863, UCB35625, and MLN-3897—have been widely explored in cancer intervention therapies with promising results. However, these inhibitors have limitations, particularly issues related to specificity. For instance, BX471 not only inhibits CCR1 but also targets CCR2, CCR5, and CXCR4, while J113863 exerts strong inhibitory effects on CCR3. In this study, we used a natural organic compound library to screen for CCR1 degraders and identified Jervine, which effectively promotes CCR1 degradation. Protein degradation in cells typically occurs *via* either the autophagic-lysosomal pathway or the ubiquitin-proteasome system. To elucidate the mechanism underlying Jervine-induced CCR1 degradation, we treated ESCC cells with MG132 (a proteasome inhibitor) and 3-MA (an autophagy inhibitor). Comprehensive analysis revealed that MG132 reverses Jervine-induced CCR1 degradation, indicating that this process depends on the ubiquitin-proteasome pathway rather than the autophagic-lysosomal pathway. Jervine is a steroidal alkaloid known for its anti-inflammatory and antioxidant properties 49. Recent studies have demonstrated its protective effects on the intestine and anticancer activity against nasopharyngeal carcinoma 49, 50. In this study, we further evaluated the *in vivo* effects of Jervine on ESCC progression and confirmed that it significantly delays tumor growth. These findings suggest that Jervine may serve as a potential candidate for CCR1-targeted degradation therapy to improve ESCC treatment outcomes.

In summary, we have confirmed the tumor-promoting role of the CCL15-CCR1 axis in ESCC and revealed the downstream AKT/ERK1/2/c-Jun/CDK2 pathway as a key regulatory mechanism. Furthermore, we identified Jervine as a novel CCR1 degrader, providing a potential candidate molecule for therapeutic intervention in ESCC. However, we acknowledge certain limitations in our study. For instance, although AKT and ERK1/2 activation correlate with c-Jun phosphorylation, they may not directly phosphorylate c-Jun, suggesting the involvement of additional kinases. The precise molecular mechanism by which Jervine induces CCR1 degradation remains to be elucidated. Moreover, the EMT process is typically accompanied by a decrease in E-cadherin expression and an increase in N-cadherin expression 51. In this study, we found that the CCL15-CCR1 axis promotes cell migration; however, N-cadherin expression was reduced, which does not align with the typical features of EMT. These aspects will be addressed in future investigations.

## Supplementary Material

Supplementary figures and tables.
