# Supplementary material for: Unlocking ESCC Progression: CCL15-CCR1 Axis Activates AKT/ERK1/2/c-Jun/CDK2 Pathway
Source: J Cancer. 2025 Jul 4;16(10):3112–27. doi: 10.7150/jca.113925 (PMC12305565; doi:10.7150/jca.113925)
Supplement: Supplementary file 1 — Supplementary figures and tables. [file jcav16p3112s1.pdf]

Supplementary materials

Table S1: The sequence of shRNA.

Target	*shRNA sequence*	*Region*	*Ref Seq*
CCR1[#1]	*CCCTACAATTTGACTATACTT*	*CDS*	*NM_001295*
CCR1[#2]	*GCTCTGAAACTGAACCTCTTT*	*CDS*	*NM_001295*
CCR1[#3]	*CCTCTGTACTCCTTGGTATTT*	*CDS*	*NM_001295*
CCL15[#1]	*CCAGTAGTTCTGAACAGCTTT*	*CDS*	*NM_004167*
CCL15[#2]	*GCACCTCCTACATCTCACAAA*	*CDS*	*NM_004167*
CCL15[#3]	*GCCCAGTTCATAAATGATGCA*	*CDS*	*NM_004167*

Table S2: The sequence of primer for RT-qPCR.

Target	*Forward Primer (5'–3')*	*Reverse Primer (5'–3')*
CCR1	*GACTATGACACGACCACAGAGT*	*CCAACCAGGCCAATGACAAATA*
CCL15	*TCCCAGGCCCAGTTCATAAAT*	*TGCTTTGTGAGATGTAGGAGGT*

Table S3: The list of antibodies for Western blotting and Immunofluorescence.

Antibody	*Manufacturer*	*Product code*
CCR1	*Abclone*	*A18341*
CCL15	*Abcam*	*Ab197016*
Ki67	*CST*	*#9449*
PCNA	*CST*	*#2586*
E-CAD	*CST*	*#14472*
N-CAD	*CST*	*#1516*
CDK2	*Santa Cruz*	*Sc-6248*
AKT	*Proteintech*	*60203-2-Ig*
p-AKT	*CST*	*#9271*
ERK1/2	*Proteintech*	*11257-1-AP*
p-ERK1/2	*CST*	*#4695*
c-Jun	*CST*	*#9165*

p-c-Jun	*CST*	*#91952*
GAPDH	*Abclone*	*AC001*

Supplementary Figure 1

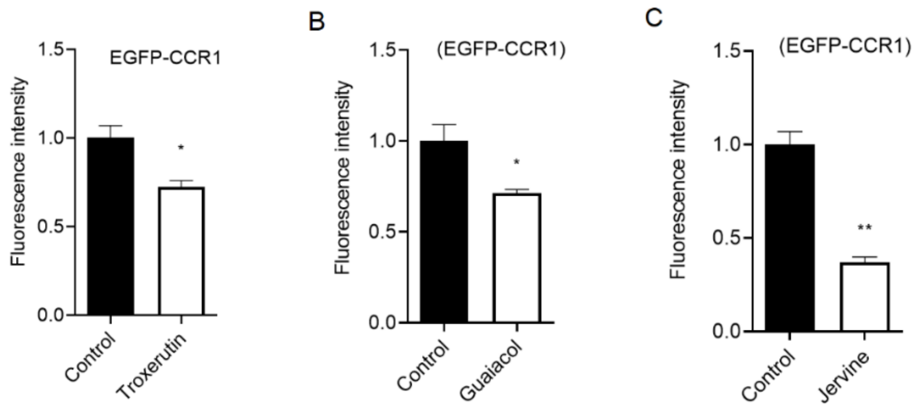

Supplementary figure 1. The degradative effects of Troxerutin, Guaiacol, and Jervine on CCR1. (A-C) The fluorescence intensity of CCR1-EGFP was measured using the fluorescence microplate reader. * vs. Control, *p<0.05, **p<0.01.

Supplementary Figure 2

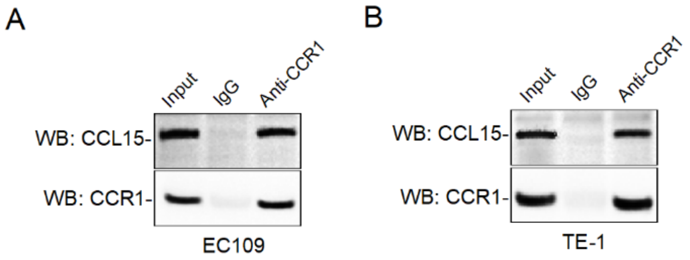

Supplementary figure 2. Interaction analysis between CCR1 and CCL15. (A-B) Interaction between CCR1 and CCL15 in EC109 and TE-1 cells was analyzed by Co-IP.

Supplementary Figure 3

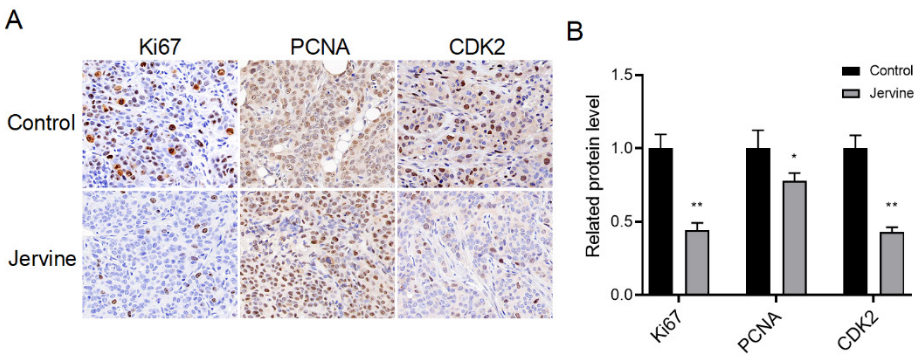

Supplementary figure 3. Effect of Jervine on the expression of Ki67, PCNA and CDK2. (A-B)

Immunohistochemistry was used to analyze Ki67, PCNA and CDK2 expressions. * vs. Control, *p<0.05, **p<0.01.
